# Bioactivity of Bioceramic Materials Used in the Dentin-Pulp Complex Therapy: A Systematic Review

**DOI:** 10.3390/ma12071015

**Published:** 2019-03-27

**Authors:** José Luis Sanz, Francisco Javier Rodríguez-Lozano, Carmen Llena, Salvatore Sauro, Leopoldo Forner

**Affiliations:** 1 Department of Stomatology, Universitat de València, 46010 Valencia, Spain; Jsanzalex96@gmail.com (J.L.S.); llena@uv.es (C.L.); 2 Cellular Therapy and Hematopoietic Transplant Unit, Hematology Department, Virgen de la Arrixaca Clinical University Hospital, IMIB, University of Murcia, 30120 Murcia, Spain; odontologofran@gmail.com; 3 School of Dentistry, Faculty of Medicine, University of Murcia, 30100 Murcia, Spain; 4 Department of Dentistry, Faculty of Health Sciences, Universidad CEU-Cardenal Herrera, 46115 Alfara del Patriarca (Valencia), Spain; salvatore.sauro@uch.ceu.es; 5 Faculty of Dentistry, Oral & Craniofacial Sciences at King’s College London, Floor 17 Tower Wing, Guy’s Hospital, London SE1 9RT, UK

**Keywords:** bioactivity, bioceramic materials, dental pulp stem cells, systematic review

## Abstract

Dentistry-applied bioceramic materials are ceramic materials that are categorized as bioinert, bioactive and biodegradable. They share a common characteristic of being specifically designed to fulfil their function; they are able to act as root canal sealers, cements, root repair or filling materials. Bioactivity is only attributed to those materials which are capable of inducing a desired tissue response from the host. The aim of this study is to present a systematic review of available literature investigating bioactivity of dentistry-applied bioceramic materials towards dental pulp stem cells, including a bibliometric analysis of such a group of studies and a presentation of the parameters used to assess bioactivity, materials studied and a summary of results. The research question, based on the PICO model, aimed to assess the current knowledge on dentistry-based bioceramic materials by exploring to what extent they express bioactive properties in in vitro assays and animal studies when exposed to dental pulp stem cells, as opposed to a control or compared to different bioceramic material compositions, for their use in the dentin-pulp complex therapy. A systematic search of the literature was performed in six databases, followed by article selection, data extraction, and quality assessment. Studies assessing bioactivity of one or more bioceramic materials (both commercially available or novel/experimental) towards dental pulp stem cells (DPSCs) were included in our review. A total of 37 articles were included in our qualitative review. Quantification of osteogenic, odontogenic and angiogenic markers using reverse transcriptase polymerase chain reaction (RT-PCR) is the prevailing method used to evaluate bioceramic material bioactivity towards DPSCs in the current investigative state, followed by alkaline phosphatase (ALP) enzyme activity assays and Alizarin Red Staining (ARS) to assess mineralization potential. Mineral trioxide aggregate and Biodentine are the prevalent reference materials used to compare with newly introduced bioceramic materials. Available literature compares a wide range of bioceramic materials for bioactivity, consisting mostly of in vitro assays. The desirability of this property added to the rapid introduction of new material compositions makes this subject a clear candidate for future research.

## 1. Introduction

Within the field of biomedical therapeutics, we can highlight the concept of tissue engineering to refer to the development of procedures and biomaterials that aim to devise new tissues to replace those damaged, following the principles of cellular and molecular biology and taking as a premise the search for “biological solutions for biological problems” [1].

In 2007, the American Association of Endodontists adopted the term “regenerative endodontics” to refer to the concept of tissue engineering applied to the restoration of root canal health, in a way that continuous development of the root and tissues surrounding it is promoted [2].

The introduction of the so-called bioceramic materials meant a great advance for this new paradigm in endodontic therapy [3], given their biocompatible nature and excellent physicochemical properties [4]. Categorized as bioinert, bioactive and biodegradable [5], dentistry-applied bioceramic materials are ceramic materials which share a common characteristic of being specifically designed to fulfil their function; they are able to act as root canal sealers, cements, root repair or filling materials [4]. Applied to vital pulp therapy, bioceramic materials can be used in cases of pulp exposition from trauma, caries or other mechanical causes, as direct pulp cappers [6].

Properties like biocompatibility and bioactivity are to be expected in dentistry-applied bioceramic materials for their use in vital pulp therapy [7]. The first one refers to the “ability to perform as a substrate that will support the appropriate cellular activity, including the facilitation of molecular and mechanical signaling systems, in order to optimize tissue regeneration, without eliciting any undesirable local or systemic responses in the eventual host” [8], while bioactivity goes even further, and is only attributed to those materials which are capable of inducing a desired tissue response from the host [9] by the use of biomimetic approaches [10]. The term differs depending on the field in which it is implemented, being related to the cellular effects induced by biologically active ions and substances released from biomaterials in the field of tissue engineering, but referred to as the biomaterial’s capability of forming hydroxyl apatite mineral on its surface both in vitro and in vivo in the field of biomaterial science [11].

Considering these desirable characteristics of bioceramic materials, it seems convenient to analyze the interaction between human dental pulp stem cells (hDPSCs), which are post-natal stem cells with mesenchymal stem cell (MSCs)-like characteristics, like auto-renewal ability and multilineage differentiation potential [12], and them; as their combined use could mean and advancement in the field of regenerative endodontics.

Cytotoxicity and biocompatibility of a wide range of bioceramic materials towards dental stem cells (DSCs) have been investigated in numerous studies [13,14,15,16,17]; among others. The well-known Pro-Root MTA (Dentsply Tulsa Dental Specialties, Tulsa, OK, USA) has been shown to increase osteoblast, fibroblast, cementoblast, odontoblast and pulp cell differentiation, but its handling difficulty among other limitations encourages for a search for alternative materials [13]. Materials like Biodentine (Septodont, Saint Maurdes-Fosses, France) and TheraCal LC (Bisco Inc., Schaumburg, IL, USA) are examples of bioceramic materials introduced posteriorly in dentistry for their use in vital pulp therapy as blood clot protectors in pulpal revascularization procedures, standing out for their consistency, easier manipulation and tricalcium silicate composition [16].

However, to the best of the authors’ knowledge, there has been no effort to sort and summarize studies analyzing bioactivity of such materials into more homogenous subgroups that would allow for an easier analysis of the evidence.

The aim of this study is to present a systematic review of available literature investigating bioactivity of dentistry-applied bioceramic materials towards dental pulp stem cells; including a bibliometric analysis of such group of studies and a presentation of the parameters used to assess bioactivity, materials studied and summary of results.

## 2. Materials and Methods

This systematic review was conducted in accordance with the PRISMA guidelines or preferred reporting items for systematic reviews and meta-analyses [18]. Our review was not eligible for registration with PROSPERO, as it did not involve health studies in which participants were people nor animal research studies exclusively.

In terms of the research question, based on the PICO model, our review aimed to assess the current knowledge on dentistry-based bioceramic materials by exploring to what extent they express bioactive properties in in vitro assays and animal studies when exposed to dental pulp stem cells, as opposed to a control or compared to different bioceramic material compositions, for their use in the dentin-pulp complex therapy.

### 2.1. Inclusion and Exclusion Criteria

Studies assessing bioactivity of one or more bioceramic materials (both commercially available or novel/experimental) towards DPSCs were included in our review. We established bioactivity assessment as any test or measurement for odontogenic, osteogenic, angiogenic and/or mineralization potential of DPSCs exposed both directly or indirectly to bioceramic materials. Studies assessing cytotoxicity and/or biocompatibility alone i.e., cell viability or proliferation were excluded. Studies assessing any other type of stem cell apart from DPSCs were also excluded.

The series of inclusion and exclusion criteria were established by a consensus reached from all authors after discussion, considering the research question and the objectives of the study while aiming for an ample range of results to be provided from the search.

### 2.2. Search Strategy

#### 2.2.1. Sources of Information

To identify potentially relevant studies, a thorough electronic search was made in PubMed, Web of Science, Scopus, Embase, Cochrane, and Lilacs databases. Study search was performed during October, November and December 2018. In particular cases, the authors of the articles were contacted by email to request missing information. The structured search strategy and data extraction were conducted by an individual examiner.

#### 2.2.2. Search Terms

**Figure 1:**
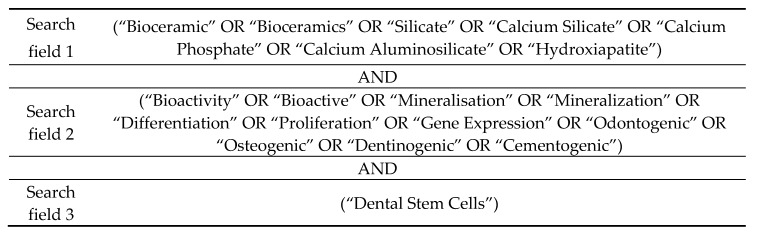
Search strategy illustration.

The search strategy included 6 Mesh (Medical Subject Heading) terms: “Silicate”, “Calcium Silicate”, “Calcium phosphate”, “Calcium aluminosilicate”, “Hydroxyapatite” and “Gene Expression”; and 13 uncontrolled descriptors: “Bioceramic”, “Bioceramics”, “Bioactivity”, “Bioactive”, “Mineralisation”, “Mineralization”, “Differentiation”, “Proliferation”, “Odontogenic”, “Osteogenic”, “Dentinogenic”, “Cementogenic” and “Dental Stem Cells”. Boolean operators (“OR” and “AND”) were used to join search terms related to the search question (Figure 1).

#### 2.2.3. Study Selection

Articles identified using the search terms were exported to RefWorks (ProQuest, MI, USA) to check for duplicates. Once duplicates were discarded, a first screening of record titles and abstracts was carried out according to the previously described inclusion and exclusion criteria. Remaining studies were assessed for eligibility and qualitative synthesis by full-text screening.

#### 2.2.4. Study Data

For the bibliometric analysis, the following variables were recorded for each article: author and year of publication, journal, country, and institution. For the synthesis of study methodology, a summary of the materials and methods of included studies was transcribed by listing the following variables: study type, bioceramic materials used, bioactivity analysis and duration of the analysis. For the synthesis of results, studies were categorized in terms of the significant results found, the duration in which these significant results were found, and their significance level.

### 2.3. Quality Assessment

The quality of the studies was assessed using a modified CONSORT checklist of items for reporting in vitro studies of dental materials [19] and the ARRIVE guidelines for reporting animal research [20].

## 3. Results

### 3.1. Study Selection and Flow Diagram

**Figure 2:**
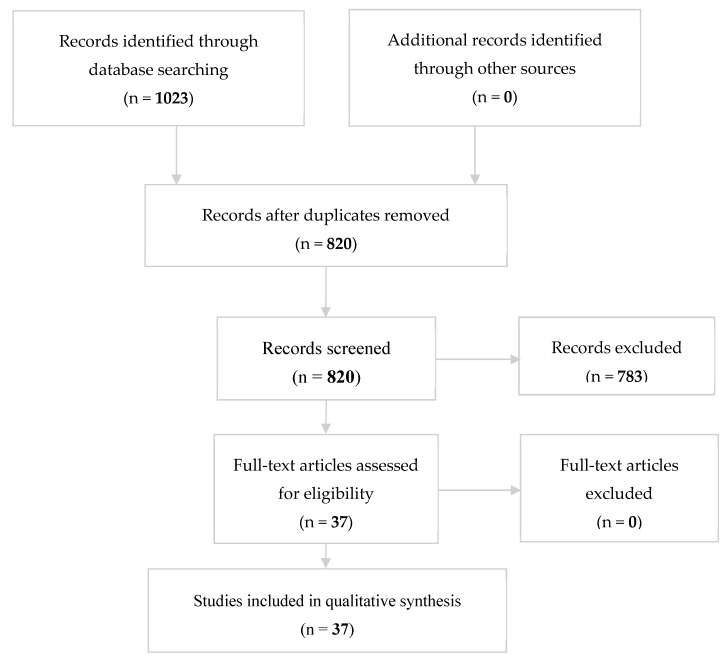
Systematic flow-chart representing study inclusion.

The search identified 1023 preliminary references related to the bioactivity of bioceramic materials towards dental stem cells, of which 355 were found in PubMed, 473 in Web of Science, 179 in Embase, 15 in Scopus, and 1 in Cochrane databases. Search made in LILACS produced no results. After excluding 203 duplicates, the remaining 820 were screened. Of these, 783 were excluded on reading the title and abstract as they did not fulfil our inclusion criteria. The resulting 37 articles were examined at full-text level, and all of them resulted to be eligible for our review (Figure 2).

### 3.2. Study Characteristics

#### 3.2.1. Bibliometric Analysis

**Figure 3:**
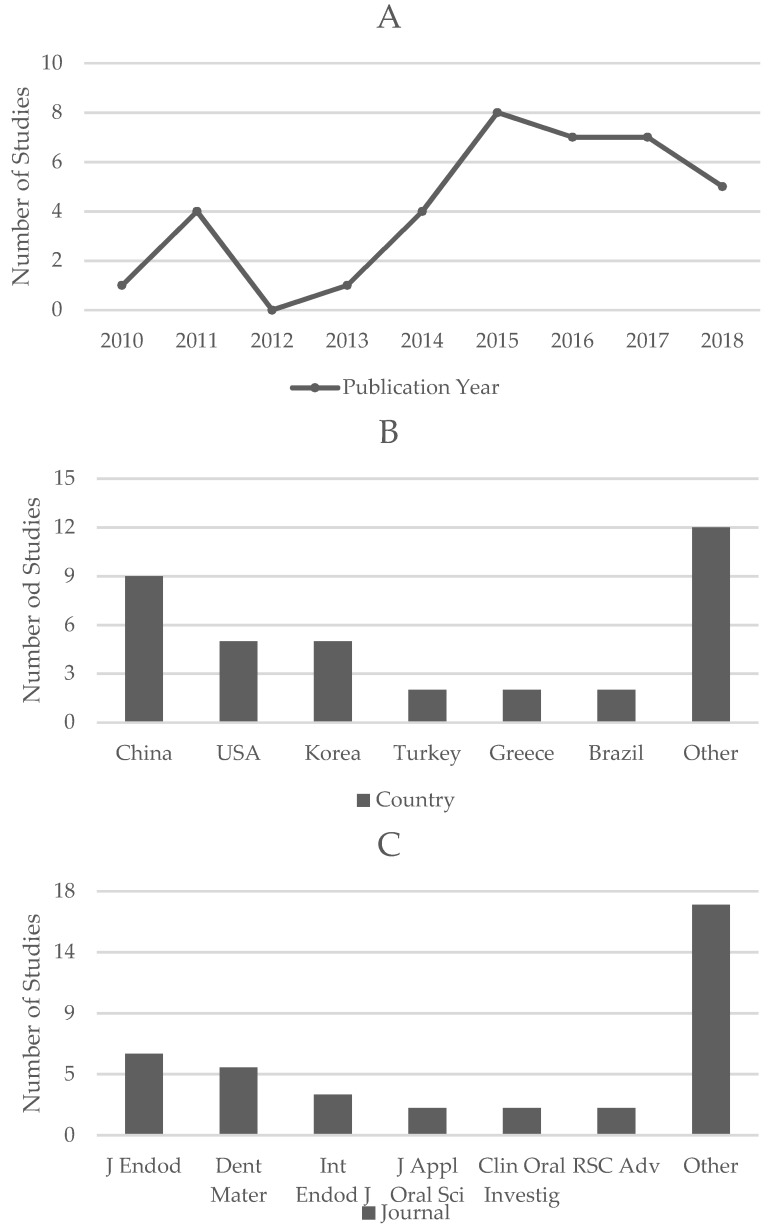
Bibliometric Analysis: distribution of included studies by year of publication (**A**), country (**B**) and journal (**C**). Studies included in the category “other” only appear once for the given bibliometric parameter.

All corresponding authors of the included studies were associated with an academic institution or university. The distribution of included studies by year of publication, country, and journal is presented in Figure 3.

#### 3.2.2. Bioactivity Analysis

A wide range of analyses of bioactivity were presented from the included studies. The most common analysis was the quantification of the expression of odontogenic, osteogenic and/or angiogenic markers or genes using reverse transcription polymerase reaction (RT-PCR), followed by alkaline phosphatase (ALP) enzyme activity assays and Alizarin Red Staining (ARS) to assess mineralization potential.

Other analyses include western blot, micro-computed tomography (micro-CT), scanning electron microscopy (SEM), attenuated total reflectance-Fourier transform infrared (ATR-FTIR), transmission electron microscopy (TEM), histological analysis, immuno-fluorescence, and immuno-histochemical assays. Bioactivity analyses alongside with their duration and a description of the study associated with them are presented in Table 1.

#### 3.2.3. Study Type

Articles included fell into two main categories in terms of type of study: in vitro, or animal study. In some cases, articles presented both an in vitro and an animal study [26,27,37]. There were two studies which analyzed bioactivity of bioceramic materials towards hDPSCs ex vivo [33,42].

#### 3.2.4. Cell Variant

All studies included used dental pulp stem cells (DPSCs) as their cell variant to assess bioceramic material bioactivity.

#### 3.2.5. Bioceramic Materials Used

Bioceramic materials studied ranged from commercially available (Table 2) to novel or experimental materials (Table 3). A separate category was presented for bioceramic materials which were combined with an additive for their analysis (Table 4).

### 3.3. Quality Assessment

All in vitro studies analyzed using the modified CONSORT checklist [19] (Table 5) presented a structured abstract (item 1) and an introduction which provided information about the background of the bioceramic material and/or bioactivity analysis studied (item 2a). Within the introduction, the majority of studies presented clear objectives and hypotheses (item 2b). Description of methodology as well as of the variables studied was sufficiently clear to allow for replication in all studies (items 3 and 4), but none of them presented a detailed report of the calculation of sample size or random allocation sequence (items 5–9). All studies indicated the statistical method used (item 10), but presented significance level as *p* values, and not confidence intervals (item 11). Discussions generally included a brief synopsis of the key findings and comparisons with relevant findings from other published studies, but often failed to address the limitations of the studies, which we considered as a reason for non-fulfillment (item 12). Sources of funding (if any) were indicated in the majority of studies (item 13), and indications for access to full trial protocols were obviated in all studies (item 14).

Only three out of the five animal studies analyzed using the ARRIVE guidelines [20] (Table 6) were headed with a sufficiently descriptive title (item 1), but all of them provided a detailed abstract (item 2). All studies provided sufficient scientific background (item 3a) and established clear objectives (item 4) in the introduction, but failed to justify the use of the animal species studied to address the scientific objectives (item 3b). Ethical statements were clear in all studies (item 5), and study design, experimental procedures were detailed enough in all except one (items 6 and 7). Details about the experimental animals and how they were distributed in the study design were included in every study (items 8–11 and 14), but housing and husbandry information was obviated in all cases (item 9). Both experimental outcomes and statistical methods were described in all studies (items 12 and 13). All studies reported the results for each analysis carried out with a measure of precision (item 15), but all of them failed to report baseline data about health status of the animals studied and any adverse effects they could have suffered after the experiment (items 14 and 17). Lastly, items referring to the discussion were fulfilled by all studies (items 18–20).

### 3.4. Study Tesults

Significant results from included in vitro studies are presented in Table 7, Table 8, Table 9, Table 10, Table 11, Table 12, Table 13, Table 14, Table 15, Table 16 and Table 17, and significant results from included animal research studies are presented in Table 18.

#### 3.4.1. Results for RT-PCR Analysis

Results for bioactivity-related marker expression using RT-PCR comparing a bioceramic material with mineral trioxide aggregate (Nex MTA/PR-MTA/MTA) showed positive significant results for the studied bioceramic materials (Exp. PPL and BD, [22]; Nano-HA, [21]), or mixed results depending on the gene/marker studied (Quick-Set2, [31]) or the concentration of material used (iRoot BP, (56]) [Table 7).

All studies comparing a bioceramic material and an additive with the bioceramic material itself showed positive significant results for the bioceramic material in combination with the additive (GNP-CPC, [25]; SC + LLLI, [30]; CPC-BGN, [32]; hTDM/SC, [33]; MTA-CaCl_2_ and MTA-Na_2_HPO_4_, [46]), except for one case (MTA+UW/PG, [35]) in which the bioceramic material itself produced better results (Table 8).

The majority of studies comparing a bioceramic material and a control showed positive significant results for the bioceramic material (Gel-HA-TCP, [23]; Zn0/1/2/3, [28]; Quick-set2 and PR-MTA, [31]; MTA and BD, [34]; BD, [38]; MTA, [40]; MTA, [43]; MTA, [44]; MTA and Theracal, [49]; MTA and BD, [50]; CaSi-αTCP, [51]; CSP, [54]; Ca_3_SiO_5_, [57]), and the rest showed mixed results depending on de gene/marker studied (Exp. PPL, BD and Nex-MTA, [22]; HA-CPC, [26]; SC, [33]; CaP, [41]; FS and BD, [48]) (Table 9).

Studies comparing a bioceramic material and a non-bioceramic material did not show positive significant results for the bioceramic materials studied. One of the studies showed that DDM produced a greater bioactivity-related gene expression than HA-CPC [26]; and the other one showed mixed results for Ca_3_SiO_3_, which produced a greater expression of some markers but not others compared to Ca(OH)_2_ [57] (Table 10).

#### 3.4.2. Results for ARS Staining

Results for ARS staining comparing a bioceramic material with mineral trioxide aggregate (MTA, PR-MTA) showed negative significant results for the studied bioceramic materials (Quick-Set2, [31]; Theracal, [49]) (Table 11).

Both studies comparing a bioceramic material and an additive with the bioceramic material itself showed positive significant results for the bioceramic material in combination with the additive (γION-CPC and αION-CPC, [24]; GNP-CPC, [25]) (Table 12).

All studies comparing a bioceramic material and a control showed positive significant results for the bioceramic materials studied (Gel-HA-TCP, [23]; Zn0/1/2/3, [28]; PR-MTA and Quick-Set2, [31]; BD, Theracal and MTA, [34]; BD, [38]; MTA, [40]; CaP, [41]; FS0.2, [48]; PR-MTA and Theracal, [49]; Ca_3_SiO_5_, [57]) (Table 13).

#### 3.4.3. Results for ALP Activity

There was only one study comparing a bioceramic material with MTA in terms of ALP activity, and it produced negative results for the bioceramic material studied (Quick-Set2, [31]). The rest of the studies compared two different biomaterials or different concentrations of the same bioceramic material (Table 14).

All studies comparing a bioceramic material and an additive with the bioceramic material itself showed positive significant results for the bioceramic material in combination with the additive (γION-CPC and αION-CPC, [24]; GNP-CPC, [25]; CPC-BGN, [32]; MTA-CaCl_2_ and MTA-NA_2_HPO_4_, [46]), except for one (SC [30]) (Table 15).

The majority of studies comparing a bioceramic material and a control showed positive significant results for the bioceramic materials studied (Gel-HA-TCP, [23]; Zn0/1/2/3, [28]; PR-MTA and Quick-Set2, [31]; SC, [33]; BD, Theracal and MTA, [34]; BD, [38]; CaP, [41]; CSC, [47]; FS and BD [48]; CSP50/100/200, [54]; Ca_3_SiO_5_, [57]). One of them showed mixed results depending on the duration of exposure (MTA, [40]) and the remaining two studies showed negative significant results for the bioceramic materials studied (MTA, [36]; MTAP and MTAF, [52]) (Table 16).

#### 3.4.4. Results for Other Bioactivity-Related Analyses

Western blot analyses showed mixed results for Zn0/1/2/3 compared to a control [28], and a higher expression of bioactivity-related markers by PR-MTA compared to Quick-Set2, and by both of them compared to a control [31]. ATR-FTIR showed positive results for PR-MTA compared to Quick-Set2, and for both of them compared to a control [31]. ELISA showed mixed results for MTA and CEM [39]. Assessment of the level of grey in mineralization nodules using Gene Tool showed positive significant results for PLGA/TCP compared to PLGA/HA and PLGA/CDHA [42]. Lastly, both the TRACP & ALP assay kit (Takahara, Shiga, Japan) and the OC and DSP emzyme-linked immunosorbent assay kit (Thermo Fisher Scientific, Waltham, MA, USA) showed that the addition of polydopamine to PR-MTA produced better results than PR-MTA itself.

## 4. Discussion

The attractiveness of bioceramic materials for their desirable properties added to their constant development, the demand for new advances and the ampliation of treatment indications results in an overflow of related literature over time. Therefore, it seems convenient to establish an updated and organized vision of the commercially available and experimental dentistry-applied bioceramic materials’ characteristics. With this in mind, the aim of this study was to present a systematic review of available literature investigating bioactivity of these materials towards dental pulp stem cells.

In terms of results, it can be highlighted that the most common method used to assess bioactivity in the included studies was the expression of bioactivity-related markers using reverse transcriptase polymerase chain reaction or RT-PCR. A recent systematic review illustrates this tendency by assessing gene expression of dental pulp cells in response to tricalcium silicate cements [58]. Studies also tended to compare new bioceramic materials with the established mineral trioxide aggregate or the more recently introduced Biodentine, as shown in Table 2, in which they appear as the most studied materials.

The use of additives in combination with bioceramic materials looks promising, in some cases enhancing or positively influencing the material’s results in bioactivity assays in comparison with the bioceramic material itself. For example, positive significant results have been shown for iron oxide [24], gold [25], and bioactive glass [32] nanoparticles in combination with calcium phosphate. However, we need to interpret these results with caution, being able to extrapolate them to clinical practice only when a clear dosage or ratio for the additive and bioceramic material has been established in controlled clinical trials.

New material compositions being studied also need to be taken into consideration for future investigations, as some of them have shown positive significant results in bioactivity assays. Novel materials like Exp. PPL [22], Gelatin-HA-TCP [23] and Zinc Bioglass (Zn0/1/2/3) [28] have all shown positive significant results for ARS staining and ALP activity assay compared to a control, and more specifically, Exp. PPL has shown a greater expression of DSPP and OCN compared to MTA and a control; Gelatin-HA-TCP has shown a greater expression of RUNX2, OSX and BSP compared to a control; and Zinc Bioglass (Zn0/1/2/3) has shown a greater expression of RUNX2, ON, CON, MEPE, BSP, and BMP-2 compared to a control. So again, in order to extrapolate these results to clinical practice, it would be interesting to carry out further studies investigating these biomaterials in different conditions.

When assessing quality and risk of bias, included studies referred a similar structural pattern. They reported essential data like a sufficient abstract, a clear objective or objectives, a detailed description of methodology, a mention of the statistical tests used and relevant conclusions; but often failed to justify the sample size used, to describe the randomization process used (if any), and most importantly to address the study’s limitations in the discussion. It may be worth noticing for future reviews that a checklist for reporting in vitro studies or “CRIS” guideline is under development [59] to address the need for uniform methodology in the assessment of this type of studies.

The introduction of new bioceramic materials and the use of additives in combination with them calls for updated research in the field. At the current state, bioactivity assessment of these materials towards dental pulp stem cells centers on in vitro assays or animal research at most. For future studies, it could be interesting to explore the mechanisms with which this bioactivity is achieved and move on towards in vivo trials.

## 5. Conclusions

Quantification of osteogenic, odontogenic and angiogenic markers using reverse transcriptase polymerase chain reaction or RT-PCR is the prevailing method used to evaluate bioceramic material bioactivity towards DPSCs in the current investigative state, followed by alkaline phosphatase (ALP) enzyme activity assays and Alizarin Red Staining (ARS) to assess mineralization potential. Mineral trioxide aggregate and Biodentine are the prevalent reference materials used to compare with newly introduced bioceramic materials. Available literature compares a wide range of bioceramic materials for bioactivity, consisting majorly of in vitro assays. The desirability of this property added to the rapid introduction of new material compositions makes this subject a clear candidate for future research.

## Figures and Tables

**Table 1:** Summary of the methodology of included studies.

Cell Variant	Study Type	Bioceramics Used	Author	Bioactivity Analysis *	Duration
hDPSCs	In vitro	MTA, Nano-HA	Hanafy et al. [21]	RT-PCR (Runx2, OCN, ALP, COL1α, OPN);	21 days
				ARS	21 days
hDPSCs	In vitro	Exp. PPL, BD, Nex-MTA	Pedano et al. [22]	RT-PCR (OCN, DSPP, ALP)	4, 10 and 14 days
hDPSCs	In vitro	Gelatin-HA-TCP (10:1:1)	Gu et al. [23]	RT-PCR (Runx2, OSX, BSP);	4, 7 and 14 days
				ALP activity;	4, 7 and 14 days
				ARS	14 and 21 days
hDPSCs	In vitro	aIONP-CPC, bIONP-CPC	Xia et al. [24]	RT-PCR (ALP, COL1α, Runx2, OCN);	7 and 14 days
				ALP activity;	4, 7 and 14 days
				ARS	7, 14 and 21 days
hDPSCs	In vitro	GNP-CPC	Xia et al. [25]	RT-PCR (ALP, COL1α, Runx2);	7 and 14 days
				ALP activity;	4, 7 and 14 days
				ARS	4, 7, 14 and 21 days
hDPCSs	In vitro, Animal	HA-TCP	Kyung-Jung et al. [26]	In vitro: RT-PCR (ALP, BSP, OPN, DMP-1, DSPP);	10 days
				In vivo: RT-PCR (BSP, OPN, ONT, OCN)	8 weeks
hDPSCs	In vitro, Animal	PCL-BCP	Wongsupa et al. [27]	RT-PCR (Runx2, ALP, OCN, DSPP);	7, 14 and 21 days
				Micro-CT;	2, 4 and 8 weeks
				Histomorphometric analysis	2, 4 and 8 weeks
hDPSCs	In vitro	Zn0, Zn1, Zn2, Zn3	Huang et al. [28]	Western blot (DSPP, DMP-1);	7 and 14 days
				RT-PCR (Runx2, OCN, BSP, BMP-2, MEPE, ON);	7 and 14 days
				ALP activity;	1, 4, 7 and 10 days
				ARS	3, 4 and 5 weeks
DPSCs	Animal	HA-TCP	Atalayin et al. [29]	RT-PCR (DSPP, DMP-1, MMP20, PHEX)	6 and 12 weeks
hDPSCs	In vitro	SC	Theocharidou et al. [30]	RT-PCR (DSPP, BMP-2, Runx2, OSX);	7 and 14 days
				ALP activity;	3, 7 and 14 days
				Mineralization analysis using SEM	28 days
hDPSCs	In vitro	Quick-Set2, PR-MTA	Niu et al. [31]	RT-PCR (Runx2, OSX, ALP, BSP, OCN, DMP-1, DSPP);	1, 2 and 3 weeks
				Western blot (DMP-1, DSPP, OCN);	1, 2 and 3 weeks
				ALP activity;	1, 2 and 3 weeks
				ARS;	1, 2 and 3 weeks
				ATR-FTIR;	1, 2 and 3 weeks
				TEM	1, 2 and 3 weeks
hDPSCs	In vitro	CPC-BGN	Lee SI et al. [32]	RT-PCR (DMP-1, DSPP, ALP, OPN, OCN, VEGF, (FGF)-2, (VEGFR)-2, VEGFR-1, (PECAM)-1, VE-cadherin;	7 and 14 days
				ALP activity;	7 and 14 days
				ARS	N/S
hDPSCs	In vitro, ex vivo	SC	Bakopoulou et al. [33]	RT-PCR (DSPP, BMP-2, Runx2, OSX, ALP, BGLAP);	7 and 14 days
				ALP activity;	3, 7 and 14 days
hDPSCs	In vitro	BD, TheraCal, MTA	Bortoluzzi et al. [34]	RT-PCR (ALP, OCN, BSP, Runx2, DSPP, DMP-1);	7 days
				ALP activity;	14 days
				ARS	14 days
hDPSCs	In vitro	MTA+UW/PG	Natu et al. [35]	RT-PCR (ALP, OCN, Runx2, DSPP, MEPE);	7 and 14 days
				ARS	7 and 14 days
hDPSCs	In vitro	BD, PR-MTA	Widbiller et al. [36]	RT-PCR (COL1α, ALP, DSPP, Runx2);	7, 14 and 21 days
				ALP activity	3, 7 and 14 days
hDPSCs	In vitro, Animal	iRoot BP Plus, PR-MTA	Zhu et al. [37]	SEM;	1, 3 and 7 days
				ATR-FTIR;	1, 3 and 7 days
				microCT;	-
				Histologic analysis;	-
				Double immunofluorescence	-
hDPSCs	In vitro	BD	Luo et al. [38]	RT-PCR (OCN, DSPP, DMP1, BSP);	14 days
				ALP activity;	1, 3, 7, 10 and 14 days
				ARS	14 days
hDPSCs	In vitro	PR-MTA, CEM	Asgary et al. [39]	RT-PCR (FGF4, BMP2, BMP4, TGF-β1, ALP, COL1, DSPP, DMP1);	1, 3, 7 and 14 days
				ELISA (FGF4, BMP2, BMP4, TGF-β1);	1, 3, 7 and 14 days
				ARS	14 days
iDPSCs	In vitro	MTA	Wang et al. [40]	RT-PCR (ALP, Runx2, OSC, OCN, DSPP);	3 and 7 days
				ALP activity;	3 and 5 days
				ARS	14 days
hDPSCs	In vitro	CaP granules	Nam et al. [41]	RT-PCR (DSPP, DMP1, COL1, OCN);	7, 14 and 21 days
				ALP activity;	7, 14 and 21 days
				ARS;	28 days
				Western blot	21 days
hDPSCs	In vitro, ex vivo	PLGA/HA, PLGA/CDHA, PLGA/TCP	Zheng et al. [42]	ALP activity;	N/S
				Von Kossa staining and Gene Tool analysis	4 and 5 weeks
hDPSCs	In vitro	MTA	Zhao et al. [43]	RT-PCR (ALP, DSPP, COL1, OCN, BSP)	6, 12, 24 and 48 h
hDPSCs	In vitro	PR-MTA	Paranjpe et al. [44]	RT-PCR (Runx2, OCN, ALP, DSP)	1, 4 and 7 days
hDPSCs	In vitro	DA0, DA0.5, DA1	Tu et al. [45]	TRACP & ALP assay kit (Takahara, Shiga, Japan);	3 and 7 days
				OC and DSP enzyme-linked immunosorbent assay kits (ThermoFisher Scientific)	7 and 14 days
hDPSCs	In vitro	PR-MTA, MTA-CaCl_2_, MTA-Na_2_HPO_4_	Kulan et al. [46]	RT-PCR (DSPP, COL1);	14 and 21 days
				ALP activity;	7 and 14 days
				Von Kossa staining	21 days
hDPSCs	In vitro	CSC	Xu et al. [47]	ALP activity	10 days
hDPSCs	In vitro	iRoot FS, BD at 0.2 and 2 mg/mL	Sun et al. [48]	RT-PCR (COL1, OCN);	1, 7 and 14 days
				ALP activity;	7 and 14 days
				ARS	21 days
hDPSCs	In vitro	TheraCal, PR-MTA	Lee BN et al. [49]	RT-PCR (DSPP, DMP1);	1 and 3 days
				ALP activity;	7 days
				ARS	14 days
hDPSCs	In vitro, Animal	BD, MTA	Daltoé et al. [50]	RT-PCR (SPP1, IBSP, DSPP, ALPL, DMP1, Runx2);	24 and 48 h
				Immunohistochemical assays for OPN y ALP;	120 days
				Indirect immunofluorescence for Runx2;	120 days
hDPSCs	In vitro	CaSi-αTCP, CaSi-DCPD	Gandolfi et al. [51]	RT-PCR (ALP, OCN)	24 h
hDPSCs	In vitro	MTAP, MTAF	Mestieri et al. [52]	ALP activity	1 and 3 days
hDPSCs	In vitro	BCP at a ratio of 20/80, 50/50 y 80/20	AbdulQader et al. [53]	RT-PCR (COL1A1, BSP, DMP1, DSPP);	14, 21 and 28 days
				ALP activity;	0–3, 3–6, 6–9, 9–12 and 12–15 days
hDPSCs	In vitro	CSP	Zhang et al. [54]	RT-PCR (DMP1, DSPP, Runx2, OPN);	3 and 10 days
				ALP activity	3 and 10 days
hDPSCs	In vitro	CPC-N, CPC-M	Lee SY et al. [55]	RT-PCR (DMP1, DSPP, OCN, OPM, BSP);	7 and 14 days
				ALP activity	7 and 14 days
hDPSCs	In vitro	iRoot BP, MTA diluted at 1:1, 1:2 o 1:5	Öncel Torun et al. [56]	RT-PCR (BMP, ON, BSP, OPN, DSPP, COL1A1, HO-1)	24 and 72 h
hDPSCs	In vitro	Ca_3_SiO_5_	Peng et al. [57]	RT-PCR (ALP, DSPP, DMP1, COL1, OC)	4, 7 and 10 days
				ALP activity;	4, 7 and 10 days
				ARS	30 days

* Genes or markers studied in RT-PCR appear inside parentheses.

**Table 2:** List of commercially available bioceramic materials studied.

Material	Abbreviation	Manufacturer	Times Studied
Mineral Trioxide Aggregate	MTA	Angelus Dental Solutions, Londrina, PR, Brazil	3
Nano-hydroxiapatite	Nano-HA	Sigma-Aldrich, UK	1
Biodentine (tricalcium silicate)	BD	Septodont, Saint Maurdes-Fosses, France	7
Nex-Cem MTA	Nex MTA	GC, Tokyo, Japan	1
Hydroxiapatite-Tricalcium Phosphate	HA-TCP	OSSTEM Implant Co., Ltd., New Zealand	1
		Zimmer, Warsaw, IN, USA	1
		N/S	1
ProRoot Mineral Trioxide Aggregate	PR-MTA	Dentsply Tulsa Dental Specialties, Tulsa, OK, USA	10
Quick-Set2	-	Primus Consulting, Bradenton, FL, USA	1
TheraCal LC	TheraCal	Bisco Inc., Schaumburg, IL, USA	2
iRoot BP Plus	-	Innovative Bioceramix, Vancouver, BC, Canada	1
Calcium-enriched mixture	CEM	BioniqueDent, Tehran, Iran	1
Hydroxyapatite	HA	N/S	1
iRoot Fast Set root repair material	FS	Innovative Bioceramix, Vancouver, BC, Canada	1
MTA Plus	MTAP	Avalon Biomed Inc., Bradenton, FL, USA	1
MTA Fillapex	MTAF	Angelus S/A, Londrina, PR, Brazil	1
FillCanal	FC	Technew, Rio de Janeiro, RJ, Brazil	1
iRoot BP	iRoot BP	Innovative Bioceramix, Vancouver, BC, Canada	1

N/S: not specified.

**Table 3:** List of experimental/novel bioceramic materials studied.

Material	Abbreviation	Composition	Times Studied
Calcium-silicate cement containing phosphopullulan	Exp. PPL	60% portland cement, 20% bismuth oxide, 5% calcium sulfate dehydrate, PPL (5%), other (10%)	1
Gelatin-hydroxyapatite-tricalcium phosphate scaffold	Gelatin-HA-TCP	Three types of powdered gelatin, HA and TCP at a ratio of 10:1:1	1
Poly-ɛ-caprolactane–biphasic calcium phosphate	PCL-BCP	80% poly-ɛ-caprolactane, 20% biphasic calcium phosphate	1
Zinc Bioglass	Zn0	38.5% SiO_2_, 26.2% Na_2_O, 29.0% CaO, 6.3% P_2_O_5_, 0% ZnO	1
	Zn1	37.0% SiO_2_, 26.5% Na_2_O, 29.2% CaO, 6.3% P_2_O_5_, 1.0% ZnO	1
	Zn2	35.7% SiO_2_, 26.7% Na_2_O, 29.4% CaO, 6.2% P_2_O_5_, 2.0% ZnO	1
	Zn3	34.3% SiO_2_, 27.0% Na_2_O, 29.6% CaO, 6.1% P_2_O_5_, 3.0% ZnO	1
Mg-based, Zn-doped bioceramic scaffolds	SC	60% SiO_2_; 7.5% MgO; 30% CaO; 2.5% ZnO	2
Calcium phosphate porous granules	CaP granules	N/S	1
Gelatin-hydroxyapatite-tricalcium phosphate	Gelatin-HA-TCP	A mixture of 3 types of powdered gelatin, HA and TCP at a ratio of 10:1:1 was added to ultrapure water to form the scaffold	1
Calcium silicate	CaSi	Dicalcium silicate, tricalcium silicate, tricalcium aluminate, calcium sulfate	1
Calcium silicate-alpha tricalcium phosphate	CaSi-αTCP	Ca_3_(PO_4_)_2_	1
Calcium silicate-dicalcium phosphate dihydrate	CaSi-DCPD	CaHPO_4_·2H_2_O	1
Hydroxyapatite-β-tricalcium phosphate	BCP	Ca_5_(PO_4_)_3_(OH)/ Ca_3_(PO_4_)_2_ at ratios of 20/80, 50/50 and 80/20	1
Silicate based Ca7Si2P2O16 bioceramic extract	CSP	Ca7Si2P2O16 diluted at a 200, 100, 50 and 25 mg/mL	1
Calcium phosphate cements in the form of nano and microparticles	CPC-N, CPC-M	α-TCP	1
Tricalcium silicate	Ca_3_SiO_5_	Ca_3_SiO_5_	1

**Table 4:** List of bioceramic materials and additives studied.

Material	Bioceramic Material Composition	Additive	Additive Composition	Abbreviation	Times Studied
Calcium phosphate cement	Tetracalcium phosphate Ca_4_(PO_4_)_2_O + dicalcium phosphate anhydrous (CaHPO_4_)	Iron oxide nanoparticles	Hematite, αFe_2_O_3_	αIONP-CPC	1
			Maghemite, βFe_2_O_3_	βIONP-CPC	
Calcium phosphate cement	Tetracalcium phosphate Ca_4_(PO_4_)_2_O + dicalcium phosphate anhydrous (CaHPO_4_)	Gold nanoparticles	Gold (III) chloride trihydrate, sodium citrate tribasic dihydrate	GNP-CPC	1
Calcium phosphate	α-tricalcium phosphate (Ca_3_(PO_4_)_2_)	Bioactive glass nanoparticles	85% SiO_2_, 15% CaO	CPC-BGN	1
Hydroxyapatite	Ca_5_(PO_4_)_3_(OH)	Poly(lactide-co-glycolide)	-	PLGA/HA	1
Hydroxiapatite-Calcium carbonate	CaCO_3_ + Ca_5_(PO_4_)_3_(OH)	Poly(lactide-co-glycolide)	-	PLGA/CDHA	1
Tricalcium phosphate	Ca_3_(PO_4_)_2_	Poly(lactide-co-glycolide)	-	PLGA/TCP	1
Mg-based, Zn-doped bioceramic scaffolds	60% SiO_2_; 7.5% MgO; 30% CaO; 2.5% ZnO	Low level laser irradiation	-	SC + LLLI	1
Premixed C_3_S/CaCl_2_ paste	C_3_S/CaCl_2_	Polyethylene glycol	-	CSC	1
ProRoot MTA	-	Propylene glycol and ultrapure water	-	MTA + UW/PG	1
ProRoot MTA	-	Polydopamine	0 mg/mL polydopamine	DA0	1
			0.5 mg/mL polydopamine	DA0.5	1
			1 mg/mL polydopamine	DA1	1
ProRoot MTA	-	Calcium chloride	CaCl_2_	MTA-CaCl_2_	1
ProRoot MTA	-	Sodium phosphate dibasic	Na_2_HPO_4_	MTA-Na_2_HPO_4_	1

**Table 5:** Results of the assessment of in vitro studies by the use of the modified CONSORT checklist [19]. Cells marked with an asterisk “*” represent study fulfilment for the given quality assessment parameter. Cells left blank represent non-fulfilment.

Studies	Modified CONSORT Checklist of Items for Reporting In Vitro Studies of Dental Materials														
	1	2a	2b	3	4	5	6	7	8	9	10	11	12	13	14
Hanafy et al. [21]	*	*		*	*						*			*	
Pedano et al. [22]	*	*	*	*	*						*		*		
Gu et al. [23]	*	*	*	*	*						*		*	*	
Xia et al. [24]	*	*	*	*	*						*			*	
Xia et al. [25]	*	*	*	*	*						*		*	*	
Kyung-Jung et al. [26]	*	*	*	*	*						*			*	
Wongsupa et al. [27]	*	*	*	*	*						*			*	
Huang et al. [28]	*	*		*	*						*				
Theocharidou et al. [30]	*	*	*	*	*						*			*	
Niu et al. [31]	*	*	*	*	*						*		*	*	
Lee SI et al. [32]	*	*		*	*						*			*	
Bakopoulou et al. [33]	*	*	*	*	*						*			*	
Bortoluzzi et al. [34]	*	*	*	*	*						*		*	*	
Natu et al. [35]	*	*	*	*	*						*			*	
Widbiller et al. [36]	*	*	*	*	*						*			*	
Zhu et al. [37]	*	*	*	*	*						*			*	
Luo et al. [38]	*	*	*	*	*						*			*	
Asgary et al. [39]	*	*	*	*	*						*			*	
Wang et al. [40]	*	*	*	*	*						*			*	
Nam et al. [41]	*	*	*	*	*						*			*	
Zheng et al. [42]	*	*		*	*						*		*	*	
Zhao et al. [43]	*	*		*	*						*			*	
Paranjpe et al. [44]	*	*	*	*	*						*		*		
Tu et al. [45]	*	*	*	*	*						*		*	*	
Kulan et al. [46]	*	*	*	*	*						*			*	
Xu et al. [47]	*	*	*	*	*						*			*	
Sun et al. [48]	*	*	*	*	*						*		*	*	
Lee BN et al. [49]	*	*	*	*	*						*			*	
Daltoé et al. [50]	*	*	*	*	*						*		*		
Gandolfi et al. [51]	*	*	*	*	*						*		*		
Mestieri et al. [52]	*	*	*	*	*						*			*	
AbdulQader et al. [53]	*	*	*	*	*						*		*	*	
Zhang et al. [54]	*	*	*	*	*						*			*	
Lee SY et al. [55]	*	*		*	*						*		*	*	
Öncel Torun et al. [56]	*	*	*	*	*						*				
Peng et al. [57]	*	*	*	*	*						*			*	

**Table 6:** Results of the assessment of animal studies by the use of the ARRIVE guidelines [20].

Studies	ARRIVE Checklist of Items for Reporting In Vivo Experiments (Animal Research)																			
	1	2	3	4	5	6	7	8	9	10	11	12	13	14	15	16	17	18	19	20
Kyung-Jung et al. [26]	*	*		*	*	*	*	*		*	*	*	*		*	*		*	*	*
Wongsupa et al. [27]	*	*		*	*	*	*	*	*	*	*	*	*	*	*	*		*	*	*
Atalayin et al. [29]		*		*	*	*	*	*		*	*	*	*		*	*		*	*	*
Zhu et al. [37]	*	*		*	*		*	*		*	*	*	*		*	*		*	*	*
Daltoé et al. [50]		*		*	*			*		*	*	*	*			*		*	*	*

**Table 7:** Summary of the results of included studies showing significant differences between various bioceramic materials or different concentrations of the same bioceramic material for osteogenic, odontogenic and/or angiogenic gene expression.

Author	Bioceramics Used	Significant Results	Gene	Duration	Significance Level
Pedano et al. [22]	Exp. PPL, BD, Nex-MTA	Exp. PPL, Biodentine > Nex MTA	DSPP	10 days	*p* < 0.05
			OCN	14 days	*p* < 0.05
		Biodentine > Nex MTA	DSPP	14 days	*p* < 0.05
Xia et al. [24]	αIONP-CPC, βIONP-CPC	γION-CPC > αION-CPC	COL1α	14 days	*p* < 0.05
Hanafy et al. [21]	MTA, Nano-HA	Nano-HA > MTA	OPN, Runx2, OCN	21 days	*p* < 0.05
Niu et al. [31]	Quick-Set2, PR-MTA	Quick-Set2 > PR-MTA	Runx2	1 and 2 weeks	*p* < 0.001
			OSX	2 and 3 weeks	*p* < 0.001
			ALP	3 weeks	*p* < 0.001
			BSP	3 weeks	*p* < 0.001
		PR-MTA > Quick-Set2	ALP	1 week	*p* < 0.001
			OCN	1, 2 and 3 weeks	*p* < 0.001
			DMP-1	1, 2 and 3 weeks	*p* < 0.001
			DSPP	2 and 3 weeks	*p* < 0.001
Sun et al. [48]	iRoot FS, BD at 0.2 and 2 mg/mL	FS0.2 > BD0.2 > BD2 > FS2	COL1	7 days	*p* < 0.05
		FS0.2 > FS2 > BD2, BD0.2	OCN	7 days	*p* < 0.05
		FS0.2 > BD0.2, BD2 > FS2	COL1	14 days	*p* < 0.05
		FS0.2, FS2 > BD0.2, BD2	OCN	14 days	*p* < 0.05
AbdulQader et al. [53]	BCP at a ratio of 20/80, 50/50 y 80/20	BCP20 > BCP50-80	DMP-1, DSPP	14, 21 and 28 days	*p* < 0.05
			BSP	21 and 28 days	*p* < 0.05
		BCP50 > BCP80	BSP	28 days	*p* < 0.05
Öncel Torun et al. [56]	iRoot BP, MTA diluted at 1:1, 1:2 o 1:5	1:1MTA > 1:1iRoot BP	OPN, DSPP	72 h	*p* < 0.05
			HO1, BMP2, BSP	24 and 72 h	*p* < 0.05
		1:1iRoot BP > 1:1 MTA	DSPP	24 h	*p* < 0.05
			ON, COL1A1	24 and 72 h	*p* < 0.05
		1:2MTA > 1:2iRoot BP	BMP2, ON, BSP	72 h	*p* < 0.05
			HO1	24 and 72 h	*p* < 0.05
		1:2iRoot BP > 1:2 MTA	OPN	24 h	*p* < 0.05
			DSPP, COL1A1	72 h	*p* < 0.05
		1:5MTA > 1:5iRoot BP	HO1, OPN, ON	72 h	*p* < 0.05
			BMP2	24 and 72 h	*p* < 0.05

**Table 8:** Summary of the results of included studies showing significant differences between a bioceramic material with an additive and the bioceramic material itself for osteogenic, odontogenic and/or angiogenic gene expression.

Author	Bioceramics Used	Significant Results	Gene	Duration	Significance Level
Xia et al. [24]	αIONP-CPC, βIONP-CPC	γION-CPC, αION-CPC > CPC	COL1α	7 days	*p* < 0.01
			ALP, Runx2	7 and 14 days	*p* < 0.01
			OCN	14 days	*p* < 0.01
Xia et al. [25]	GNP-CPC	GNP-CPC > CPC	COL1α, ALP, Runx2	7 and 14 days	*p* < 0.01
			OCN	14 days	*p* < 0.01
Theocharidou et al. [30]	SC	SC + low level laser treatment > SC	DSPP, BMP-2, OSX, Runx2	7 and 14 days	*p* < 0.05
Lee SI et al. [32]	CPC-BGN	CPC-BGN10% > CPC-BGN5% > CPC-BGN2%	OPN, DSPP, FGF2, VEGF, PECAM-1	7 and 14 days	*p* < 0.05
			VEGFR1	14 days	*p* < 0.05
		CPC-BGN10% > CPC-BGN2%	DMP-1, VEGFR2. VE-cadherin	7 and 14 days	*p* < 0.05
		CPC-BGN2, 5, 10% > CPC-BGN0%	PECAM-1, DSPP	7 and 14 days	*p* < 0.05
			ALP, OPN, DMP-1, VEGF, VEGFR1, VE-cadherin	14 days	*p* < 0.05
		CPC-BGN5, 10% > CPC-BGN0%	FGF2	7 and 14 days	*p* < 0.05
			OPN, VEGF	7 days	*p* < 0.05
			OCN	14 days	*p* < 0.05
		CPC-BGN10% > CPC-BGN0%	VEGFR2	7 and 14 days	*p* < 0.05
			DMP-1, VE-cadherin	7 days	*p* < 0.05
Bakopoulou et al. [33]	SC	hTDM/SC > SC	BMP-2	7 and 14 days	*p* < 0.05
			DSPP	14 days	*p* < 0.05
		SC > hTDM	Runx2	7 days	*p* < 0.05
Natu et al. [35]	MTA + UW/PG	MTA + UW/PG (100/0) > MTA + UW/PG (50/50)	OCN, DSPP	14 days	*p* < 0.05
Kulan et al. [46]	PR-MTA, MTA-CaCl_2_, MTA-Na_2_HPO_4_	MTA-CaCl_2_, MTA-Na_2_HPO_4_ > PR-MTA + distilled water	COL1, DSPP	14 and 21 days	*p* < 0.05

**Table 9:** Summary of the results of included studies showing significant differences between a bioceramic material and a control for osteogenic, odontogenic and/or angiogenic gene expression.

Author	Bioceramics Used	Significant Results	Gene	Duration	Significance Level
Pedano et al. [22]	Exp. PPL, BD, Nex-MTA	Biodentine, Exp. PPL, Nex MTA < control	ALP	4 and 14 days	*p* < 0.05
			DSPP, OCN	4 days	*p* < 0.05
		Biodentine > control	DSPP, OCN	10 days	*p* < 0.05
		Biodentine, Ex. PPL > control	OCN	14 days	*p* < 0.05
Gu et al. [23]	Gelatin-HA-TCP (10:1:1)	Gel-HA-TCP > control	Runx2	4 days	*p* < 0.01
			OSX	7 days	*p* < 0.05
			BSP	4 days	*p* < 0.05
Kyung-Jung et al. [26]	HA-TCP	HA-CPC > control	ALP	10 days	*p* < 0.05
			BSP	10 days	*p* < 0.001
		Control > HA-CPC	OPN, DMP-1	10 days	*p* < 0.01
			DSPP	10 days	*p* < 0.001
Huang et al. [28]	Zn0, Zn1, Zn2, Zn3	Zn1, Zn2 > control	Runx2	7 days	*p* < 0.01
		Zn0 > control		14 days	*p* < 0.05
		Zn1, Zn2, Zn3 > control		14 days	*p* < 0.01
		Zn1 > control	ON	7 days	*p* < 0.05
		Zn0, Zn1, Zn2, Zn3 > control		14 days	*p* < 0.05
		Zn0 > control	OCN	7 days	*p* < 0.01
		Zn3 > control		14 days	*p* < 0.05
		Zn0, Zn1, Zn2, Zn3 > control		14 days	*p* < 0.01
		Zn0 > control	MEPE	7 days	*p* < 0,05
		Zn1, Zn2, Zn3 > control		7 days	*p* < 0.01
		Zn0, Zn1, Zn2, Zn3 > control		14 days	*p* < 0.01
		Zn0, Zn1, Zn2, Zn3 > control	BSP	7 days	*p* < 0.01
		Zn0 > control		14 days	*p* < 0.05
		Zn1, Zn2, Zn3 > control		14 days	*p* < 0.01
		Zn1, Zn2, Zn3 > control	BMP-2	7 days	*p* < 0.05
		Zn0, Zn2, Zn3 > control		14 days	*p* < 0.01
		Zn1 > control		14 days	*p* < 0.05
Bakopoulou et al. [33]	SC	SC > control	DSPP, BMP-2, BGLAP	7 and 14 days	*p* < 0.05
			OSX	14 days	*p* < 0.05
		Control > SC	ALP	7 and 14 days	*p* < 0.05
			Runx2	14 days	*p* < 0.05
Niu et al. [31]	Quick-Set2, PR-MTA	Quick-Set2, PR-MTA > control	Runx2	1 and 2 weeks	*p* < 0.001
			OSX, DSPP	2 and 3 weeks	*p* < 0.001
			ALP	1 and 3 weeks	*p* < 0.001
			BSP	3 weeks	*p* < 0.001
			OCN, DMP-1	1, 2 and 3 weeks	*p* < 0.001
Bortoluzzi et al. [34]	BD, TheraCal LC, MTA	Biodentine, MTA > control	ALP, OCN, BSP, DSPP, DMP-1	7 days	*p* < 0.0085
Luo et al. [38]	BD	Biodentine 0.2 mg/mL, Biodentine 2 mg/mL > control	OCN, DSPP, DMP1, BSP	14 days	*p* < 0.05
Wang et al. [40]	MTA	MTA > control	OCN	3 days	P < 0.05
			Runx2, OSX, DSPP	3 and 7 days	*p* < 0.01
			ALP, OCN	7 days	*p* < 0.01
Nam et al. [41]	CaP granules	CaP > control	DSPP, DMP1, OCN	21 days	*p* < 0.01
			COL1	14 days	*p* < 0.05
		CaP > control	COL1, OCN, DSPP	7 days	*p* < 0.01
			DMP1	14 days	*p* < 0.05
Zhao et al. [43]	MTA	MTA > control	ALP, DSPP, COL1, BSP	6, 12, 24 and 48 h	*p* < 0.05
			OCN	12, 24 and 48 h	*p* < 0.05
		MTA 0.2 mg/mL, MTA 2 mg/mL > control	ALP, DSPP, COL1, BSP, OCN	48 h	*p* < 0.05
Paranjpe et al. [44]	MTA	MTA > control	OCN, ALP, DSP	7 days	*p* < 0.05
			Runx2	4 days	*p* < 0.05
Sun et al. [48]	iRoot FS, BD at 0.2 and 2 mg/mL	Control > BD2	COL1	1 and 7 days	*p* < 0.05
			OCN	7 days	*p* < 0.05
		Control > BD0.2, FS0.2, FS2	COL1	7 days	*p* < 0.05
		FS0.2 > control	OCN	7 days	*p* < 0.05
			COL1	14 days	*p* < 0.05
Lee BN et al. [49]	TheraCal, PR-MTA	PR-MTA > control	DSPP	1 and 3 days	*p* < 0.05
			DMP	3 days	*p* < 0.05
		Theracal > control	DSPP, DMP	3 days	*p* < 0.05
Daltoé et al. [50]	BD, MTA	Biodentine, MTA > control	SPP1, ALPL, Runx2	48 h	*p* < 0.05
Gandolfi et al. [51]	CaSi-αTCP, CaSi-DCPD	CaSi-αTCP > control	ALP, OCN	24 h	*p* < 0.05
Zhang et al. [54]	CSP diluted at 200, 100, 50 y 25 mg/mL	CSP25, CSP50, CSP100, CSP200 > control	DSPP, DMP1	10 days	*p* < 0.05
			Runx2	3 and 10 days	*p* < 0.05
		CSP50, CSP100, CSP200 > control	DSPP, DMP1, OPN	3 days	*p* < 0.05
		CSP100, CSP200 > control	OPN	10 days	*p* < 0.05
Peng et al. [57]	Ca_3_SiO_5_	Ca_3_SiO_5_ > control	ALP, DSPP	4, 7 and 10 days	*p* < 0.05
			OC, DMP1	7 and 10 days	*p* < 0.05

**Table 10:** Summary of the results of included studies showing significant differences between a bioceramic material and a non-bioceramic material for osteogenic, odontogenic and/or angiogenic gene expression.

Author	Bioceramics Used	Other Material Used	Bioactivity Analysis	Significant Results	Gene	Duration	Significance Level
Kyung-Jung et al. [26]	HA-TCP	Demineralized dentin matrix	RT-PCR (ALP, BSP, OPN, DMP-1, DSPP)	DDM > HA-CPC	ALP, BSP, OPN	10 days	*p* < 0.05
					DMP-1	10 days	*p* < 0.01
					DSPP	10 days	*p* < 0.001
Peng et al. [57]	Ca_3_SiO_5_	Calcium Hydroxide (Ca(OH)_2_)	RT-PCR (ALP, COL1, OC, DSPP, DMP1)	Ca_3_SiO_5_ > Ca(OH)_2_	ALP, DSPP	4, 7 and 10 days	*p* < 0.05
				Ca(OH)_2_ > Ca_3_SiO_5_	OC, DMP1	7 and 10 days	*p* < 0.05
					DMP1	4 days	*p* < 0.05

**Table 11:** Summary of the results of included studies showing significant differences between various bioceramic materials or different concentrations of the same bioceramic material for ARS staining.

Author	Bioceramics Used	Significant Results	Duration	Significance Level
Xia et al. [24]	αIONP-CPC, βIONP-CPC	γION-CPC > αION-CPC	14 and 21 days	*p* < 0.05
Niu et al. [31]	Quick-Set2, PR-MTA	PR-MTA > Quick-Set2	2 and 3 weeks	*p* < 0.001
Lee BN et al. [49]	TheraCal, PR-MTA	PR-MTA > Theracal	14 days	*p* < 0.05

**Table 12:** Summary of the results of included studies showing significant differences between a bioceramic material with an additive and the bioceramic material itself for ARS staining.

Author	Bioceramics Used	Significant Results	Duration	Significance Level
Xia et al. [24]	αIONP-CPC, βIONP-CPC	γION-CPC, αION-CPC > CPC	7 and 14 days	*p* < 0.05
Xia et al. [25]	GNP-CPC	GNP-CPC > CPC	14 and 21 days	*p* < 0.01

**Table 13:** Summary of the results of included studies showing significant differences between a bioceramic material and a control for ARS staining.

Author	Bioceramics Used	Significant Results	Duration	Significance Level
Gu et al. [23]	Gelatin-HA-TCP (10:1:1)	Gel-HA-TCP > control	18 days	*p* < 0.01
			21 days	*p* < 0.05
Huang et al. [28]	Zn0, Zn1, Zn2, Zn3	Zn0, Zn1, Zn2, Zn3 > control	3 weeks	*p* < 0.05
		Zn1, Zn2, Zn3 > control	4 and 5 weeks	*p* < 0.05
Niu et al. [31]	Quick-Set2, PR-MTA	PR-MTA, Quick-Set2 > control	1, 2 and 3 weeks	*p* < 0.001
Bortoluzzi et al. [34]	BD, TheraCal, MTA	Biodentine, TheraCal, MTA > control	7 and 14 weeks	*p* < 0.05
Luo et al. [38]	BD	Biodentine 0.2 mg/mL, Biodentine 2 mg/mL > control	14 days	*p* < 0.05
Wang et al. [40]	MTA	0.2 mg/mL MTA > control	14 days	*p* < 0.01
Nam et al. [41]	CaP granules	CaP > control	28 days	*p* < 0.01
Sun et al. [48]	iRoot FS, BD at 0.2 and 2 mg/mL	FS0.2 > control	21 days	*p* < 0.05
Lee BN et al. [49]	TheraCal, PR-MTA	PR-MTA, Theracal > control	14 days	*p* < 0.05
Peng et al. [57]	Ca_3_SiO_5_	Ca_3_SiO_5_ > control	30 days	*p* < 0.05

**Table 14:** Summary of the results of included studies showing significant differences between various bioceramic materials or different concentrations of the same bioceramic material for ALP activity.

Author	Bioceramics Used	Significant Results	Duration	Significance Level
Xia et al. [24]	αIONP-CPC, βIONP-CPC	γION-CPC > αION-CPC	14 days	*p* < 0.05
Niu et al. [31]	Quick-Set2, PR-MTA	PR-MTA > Quick-Set2	2 and 3 weeks	*p* < 0.001
Zheng et al. [42]	PLGA/HA, PLGA/CDHA, PLGA/TCP	PLGA/TCP > PLGA/HA, PLG/CDHA	N/S	*p* < 0.05
Sun et al. [48]	iRoot FS, BD at 0.2 and 2 mg/mL	FS0.2, FS2, BD0.2 > BD2	7 days	*p* < 0.05
		FS0.2 > BD0.2 > BD2, FS2	14 days	*p* < 0.05
Mestieri et al. [52]	MTAP, MTAF	MTAP > MTAF	1 y 3 days	*p* < 0.05
AbdulQader et al. [53]	BCP at a ratio of 20/80, 50/50 y 80/20	BCP20 > BCP50, BCP20	3–6, 6–9, 9–12 and 12–15 days	*p* < 0.05
		BCP50 > BCP80	9–12 and 12–15 days	*p* < 0.05
Lee SY et al. [55]	CPC-N, CPC-M	CPC-N > CPC-M	7 and 14 days	*p* < 0.05

**Table 15:** Summary of the results of included studies showing significant differences between a bioceramic material with an additive and the bioceramic material itself for ALP activity.

Author	Bioceramics Used	Significant Results	Duration	Significance Level
Xia et al. [24]	αIONP-CPC,βIONP-CPC	γION-CPC, αION-CPC > CPC	7 days	*p* < 0.05
			14 days	*p* < 0.01
Xia et al. [25]	GNP-CPC	GNP-CPC > CPC	7 and 14 days	*p* < 0.01
Theocharidou et al. [30]	SC	SC + LLLI > SC	7 days	*p* < 0.05
		SC > SC + LLLI	14 days	*p* < 0.05
Lee SI et al. [32]	CPC-BGN	CPC-BGN2, 5, 10% > CPC-BGN0%	7 and 14 days	*p* < 0.05
Kulan et al. [46]	PR-MTA, MTA-CaCl_2_, MTA-Na_2_HPO_4_	MTA-CaCl_2_, MTA-Na_2_HPO_4_ > PR-MTA + distilled water	N/S	*p* < 0.01

**Table 16:** Summary of the results of included studies showing significant differences between a bioceramic material and a control for ALP activity.

Author	Bioceramics Used	Significant Results	Duration	Significance Level
Gu et al. [23]	Gelatin-HA-TCP (10:1:1)	Gel-HA-TCP > control	4 days	*p* < 0.05
			7 and 12 days	*p* < 0.01
Huang et al. [28]	Zn0, Zn1, Zn2, Zn3	Zn3 > control	1 day	*p* < 0.05
		Zn0, Zn1, Zn2, Zn3 > control	7 and 10 days	*p* < 0.05
Niu et al. [31]	Quick-Set2, PR-MTA	PR-MTA, Quick-Set2 > control	1, 2 and 3 weeks	*p* < 0.001
Bakopoulou et al. [33]	SC	SC > control	3, 7 and 14 days	*p* < 0.05
Bortoluzzi et al. [34]	BD, TheraCal, MTA	Biodentine, MTA, TheraCal > control	14 days	*p* < 0.05
Widbiller et al. [36]	BD, PR-MTA	Control > MTA	3, 7 and 14 days	*p* < 0.05
Luo et al. [38]	BD	Biodentine 0.2 mg/mL, Biodentine 2 mg/mL > control	7, 10 and 14 days	*p* < 0.05
		Biodentine 0.2 mg/mL > control	3 days	*p* < 0.05
Wang et al. [40]	MTA	0.02 mg/mL MTA > control	3 days	*p* < 0.05
		0.2 mg/mL MTA > control	3 and 5 days	*p* < 0.01
		2 mg/mL MTA > control	5 days	*p* < 0.01
		Control > 20 mg/mL MTA	3 and 5 days	*p* < 0.01
Nam et al. [41]	CaP granules	CaP > control	14 and 21 days	*p* < 0.01
Xu et al. [47]	CSC	CSC > control	10 days	*p* < 0.05
Sun et al. [48]	iRoot FS, BD at 0.2 and 2 mg/mL	BD0.2, BD2, FS0.2, FS2 > control	7 and 14 days	*p* < 0.05
Lee BN et al. [49]	TheraCal, PR-MTA	MTA > control	7 days	*p* < 0.05
Mestieri et al. [52]	MTAP, MTAF	Control > MTAP, MTAF	1 and 3 days	*p* < 0.05
Zhang et al. [54]	CSP diluted at 200, 100, 50 y 25 mg/mL	CSP50, CSP100, CSP200 > control	3 and 10 days	*p* < 0.05
Peng et al. [57]	Ca_3_SiO_5_	Ca_3_SiO_5_ > control	10 days	*p* < 0.05

**Table 17:** Summary of the results of included studies showing significant differences for another bioactivity-related analysis.

Author	Bioceramics Used	Bioactivity Analysis	Significant Results	Gene	Duration	Significance Level
Huang et al. [28]	Zn0, Zn1, Zn2, Zn3	Western Blot	Zn0, Zn1, Zn2, Zn3 > control	DSPP	7 days	*p* < 0.05
			Control > Zn0, Zn1, Zn2, Zn3	DSPP	14 days	*p* < 0.05
			Zn2, Zn3 > control	DMP-1	7 days	*p* < 0.05
			Control > Zn0, Zn1, Zn2, Zn3	DSPP	14 days	*p* < 0.05
Niu et al. [31]	Quick-Set2, PR-MTA	Western Blot	PR-MTA > Quick-Set2	DMP-1 DSPP, OCN	1, 2 and 3 weeks 2 and 3 weeks	*p* < 0.001 *p* < 0.001
			PR-MTA, Quick-Set2 > control	DMP-1 DSPP	1, 2 and 3 weeks 2 and 3 weeks	*p* < 0.001 *p* < 0.001
			PR-MTA > control	OCN	1, 2 and 3 weeks	*p* < 0.001
		ATR-FTIR	PR-MTA > Quick-Set2	-	2 and 3 weeks	*p* < 0.001
			PR-MTA, Quick-Set2 > control	-	1, 2 and 3 weeks	*p* < 0.001
Asgary et al. [39]	MTA, CEM	ELISA	MTA > CEM	TFG-β1	N/S	*p* < 0.05
			CEM > MTA	FGF4	N/S	*p* < 0.05
Zheng et al. [42]	PLGA/HA, PLGA/CDHA, PLGA/TCP	Gene Tool (level of grey in mineralization nodules analysis)	PLGA/TCP > PLGA/HA, PLG/CDHA	-	4 weeks	*p* < 0.05
			PLGA/TCP > PLGA/HA	-	5 weeks	*p* < 0.05
Tu et al. [45]	DA0, DA0.5, DA1	TRACP & ALP assay kit (Takahara, Shiga, Japan)	DA0.5, DA1 > DA0	ALP	7 days	*p* < 0.05
			DA1 > DA0	ALP	3 days	*p* < 0.05
			DA0.5, DA1 > DA0	OCN	7 and 14 days	*p* < 0.05
		OC and DSP enzyme-linked immunosorbent assay kits (ThermoFisher Scientific)	DA1 > DA0	DSP	7 days	*p* < 0.05
			DA0.5, DA1 > DA0	DSP	14 days	*p* < 0.05

**Table 18:** Summary of the significant results of included studies categorized as animal studies.

Author	Bioceramics Used	Non-Bioceramic Material Used	In Vivo Assay Description	Bioactivity Analysis	Results	Gene	Duration	Significance Level
Kyung-Jung et al. [26]	HA-TCP	Demineralized dentin matrix	Ectopic bone fomation in athymic rats with HA-(HA-TCP or DDM) and HDPSCs implanted subcutaneously.	RT-PCR	HA-TCP > DDM	BSP	8 weeks	*p* < 0.001
Wongsupa et al. [27]	PCL-BCP	-	Bone formation in 1mm diameter calvarial defects in the parietal bone in 5–6-month-old male New Zealand white rabbits.	Micro-CT	PCL-BCP + hDPSCs > PCL-BCP	-	4 and 8 weeks	*p* < 0.01
				Hysto-morphometric anaysis	PCL-BCP + hDPSCs > PCL-BCP	-	2, 4 and 8 weeks	*p* < 0.01
Atalayin et al. [29]	HA-TCP	L-lactide/DL-lactide copolymer (PLDL), DL-lactide copolymer (PDL)	Odontogenic differentiation of hDPSCs in rats implanted with HA-CPC and hDPSCs subcutaneously.	RT-PCR (DSPP, DMP-1, MMP20, PHEX)	PDL > PLDL, HA/TCP	DSPP	12 weeks	*p* < 0.05
					PLDL > PDL, HA-TCP	DMP1	6 weeks	*p* < 0.05
					HA-TCP > PLDL, PDL		12 weeks	*p* < 0.05
					PLDL, HA/TCP > PDL	MMP20	6 weeks	*p* < 0.05
					PDL > PLDL, HA/TCP		12 weeks	*p* < 0.05
					HA-TCP > PLDL, PDL	PHEX		*p* < 0.05
					PLDL > PDL, HA-TCP			*p* < 0.05
Daltoé et al. [50]	BD, MTA	-	87 specimens of 2nd and 3rd upper premolars and 2nd, 3rd, and 4th lower premolars from 4 dogs (Beagles), evaluated 120 days after pulpotomy.	Staining for OPN and ALP in mineralized tissue bridge	MTA > BD	OPN	120 days	*p* < 0.001
					BD > MTA	ALP	120 days	*p* < 0.001
				Staining for OPN and ALP in pulp tissue	BD > MTA	OPN	120 days	*p* < 0.001
